# Detecting early kidney injury due to host–virus interaction response in treatment-naive CHB—a pilot study

**DOI:** 10.3389/fcimb.2025.1601678

**Published:** 2025-07-29

**Authors:** Puneet Gandhi, Kavita Peter, Subodh Varshney, Mahendra Kumar Atlani, Kewal Krishan Maudar

**Affiliations:** ^1^ Department of Research and Training, Bhopal Memorial Hospital and Research Centre, Bhopal, India; ^2^ Department of Gastrointestinal Surgery, Bhopal Memorial Hospital and Research Centre, Bhopal, India; ^3^ Siddhanta Red Cross Superspeciality Hospital, Bhopal, India; ^4^ Department of Nephrology, Bhopal Memorial Hospital and Research Centre, Bhopal, India; ^5^ Department of Nephrology, All India Institute of Medical Science, Bhopal, India; ^6^ Faculty, Bharati Vidyapeeth Medical College, Pune, India

**Keywords:** biomarker, kidney injury, hepatitis B virus, chronic hepatitis B, inflammation

## Abstract

**Purpose:**

Clinical evidence suggests that patients with chronic hepatitis B (CHB) have an increased risk of renal impairment due to inflammation induced by virus–host interaction. We aimed to evaluate and validate a set of protein biomarkers singularly and in combination for the early detection of subclinical kidney injury in patients with CHB naive to antiretroviral therapy.

**Methods:**

This work is part of a prospective cross-sectional study for which 69 HBsAg-positive, treatment-naive patients with CHB with an equal number of age-matched healthy volunteers were considered. At diagnosis, serum creatinine (sCr), urea, alanine transaminase, aspartate transaminase, serum cystatin-C (sCys-C), serum neutrophil gelatinase-associated lipocalin (sNGAL), serum Fetuin-A (sFet-A), urinary interleukin-18 binding protein (uIL-18BP), and urinary kidney injury molecule-1 (uKIM-1) levels were determined.

**Results:**

There was a significant elevation in the concentrations of three proteins in our CHB cohort (sCys-C, sNGAL, and uIL-18BP; *p* < 0.0001) while sFet-A was down-regulated (*p*<0.01) as compared to the control group. A receiver operating characteristic curve analysis revealed an Area under the curve of 0.935 for sCys-C and 0.811 for sNGAL, which improved to 0.984 when all four indicators were combined in a panel to discriminate the onset of renal injury incited by inflammatory response in CHB with 97.1% sensitivity at 91.3% specificity. Additionally, only sCys-C and sNGAL differed significantly among the phases of CHB infection (*p*<0.05).

**Conclusions:**

This novel noninvasive diagnostic screen is expedient in detecting inflammation and early kidney injury before a rise in sCr and can aid in predicting renal outcomes in patients with CHB.

## Introduction

Hepatitis B infection has become a global health concern due to its adverse outcomes, including liver cirrhosis, hepatocellular carcinoma, and renal impairment. According to the latest World Health Organization (WHO) estimates, approximately 1.1 million people died due to hepatitis B-related complications in 2022 (World Health Organization, 2024). India presented the second highest viral hepatitis cases, which account for 11.7% of the total global burden. Also, India, together with China and Indonesia, showed 50% of the global viral hepatitis load (World Health Organization, 2024).

Chronic hepatitis B virus (HBV) infection is a major etiological factor in an array of renal diseases, with co-existent clinical comorbidities, which altogether leads to a worse prognosis (Liu et al., 2025; Cacoub and Asselah, 2022). The underlying mechanism of HBV evasion remains controversial, but different groups suggest that the virus dodges recognition and escapes the host’s innate and adaptive immune responses (Zhao et al., 2022; Kuipery et al., 2020). HBV mediates kidney damage in the host via immune-complex deposition in the glomerulus, activating complement‐mediated inflammatory response leading to apoptosis in tubular epithelial cells (Yu et al., 2022). The ability to improve clinical outcomes in chronic hepatitis B (CHB) is hindered by insufficient lab support to diagnose renal involvement at a reversible stage early in the disease process. To date, numerous biochemical markers have been assessed in cohorts with defined extrahepatic kidney ailments. They have changed our outlook towards serum creatinine (sCr) and serum urea (sUr), as these conventional parameters take weeks to escalate after a renal insult, especially in cases where the primary diagnosis is a virus-induced liver ailment. The current developments in molecular proteomics have greatly augmented the discovery of novel noninvasively evaluated markers that promptly fluctuate in body fluids in response to viral stimuli.

**Figure 1:**
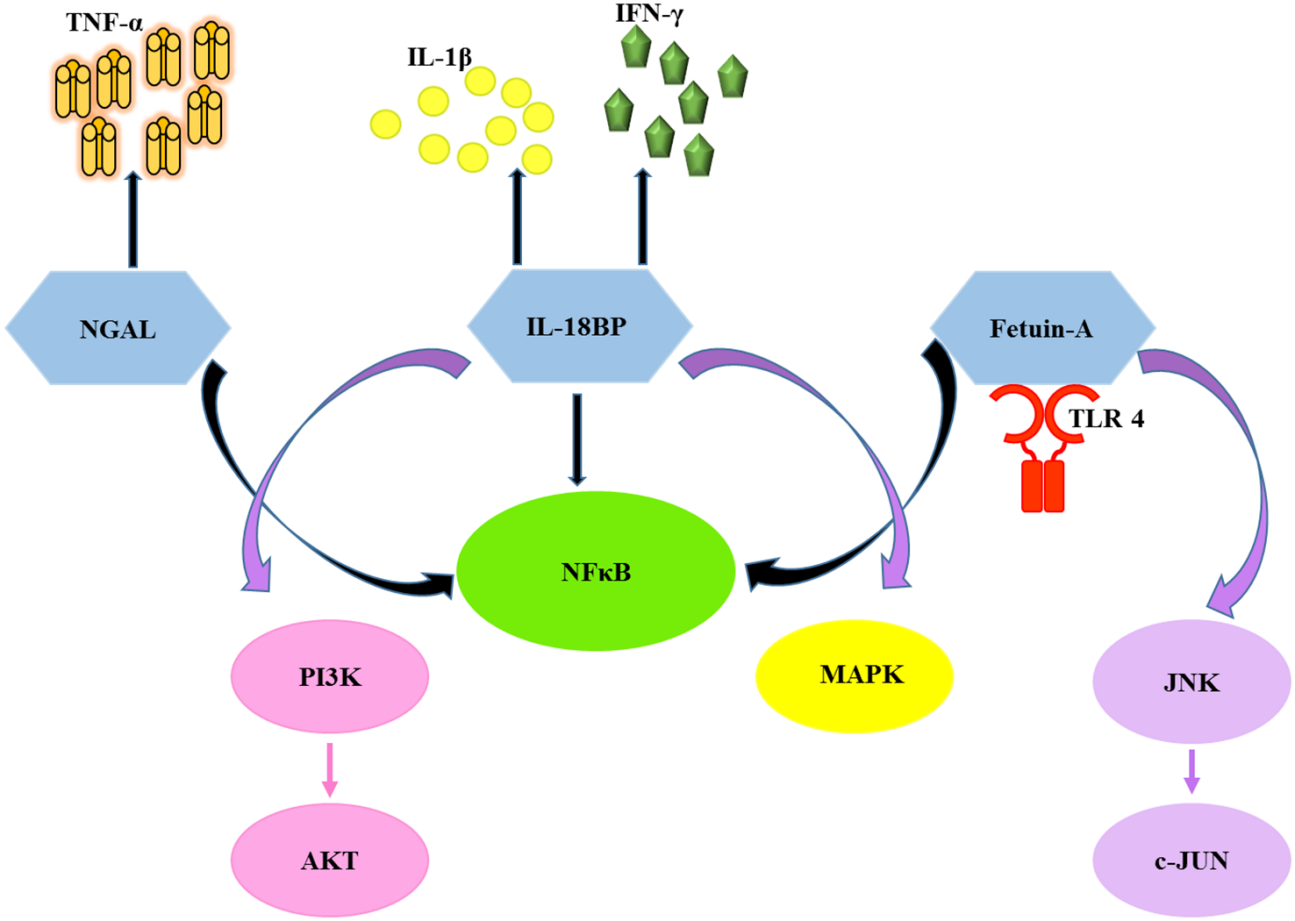
Diagram illustrates the activation of pro-inflammatory cytokines and related pathways by the chosen biomarkers. NGAL, neutrophil gelatinase associated lipocalin; Fet-A, fetuin-A; IL-18BP, interleukin-18 binding protein; TLR4, toll-like receptor 4; NFκB, nuclear factor kappa-light-chain-enhancer of activated B cells; JNK, c-Jun N-terminal kinase; c-JUN, c-Jun proto-oncogene; MAPK, mitogen-activated protein kinase; PI3K, phosphatidylinositol 3-kinase; AKT, protein kinase B (PKB); IL-1β, interleukin-1 beta; IFN-γ, interferon gamma; TNF-α,tumor necrosis factor alpha.

Kidney diseases are multifactorial and heterogeneous in origin; therefore, it is likely that not one single biomarker but a panel of biomarkers representing all aspects of renal involvement may better detect kidney injury during early disease onset. Cystatin-C (Cys-C; 13 kDa) is an inhibitor of the cysteine protease and a marker of renal function. It is produced by nucleated cells and catabolized by tubular epithelial cells (Fiseha, 2015). Circulating neutrophil gelatinase-associated lipocalin (NGAL; 25 kDa), a protein predominantly associated with human neutrophil gelatinase, is a promising early diagnostic biomarker for acute kidney injury (AKI) (Romejko et al., 2023). KIM-1 is a type I transmembrane glycoprotein (90 kDa), a marker of proximal tubular injury. It is undetectable in healthy kidney tissue but upregulated in various human kidney diseases (Anandkumar et al., 2023). Fet-A is a negative acute-phase reactant glycoprotein (64 kDa) exclusively synthesized by the liver and is downregulated in systemic inflammation (Ricken et al., 2022). IL-18BP is an anti-inflammatory secretory protein with a high affinity for IL-18 (a marker for renal damage), which inhibits the production of interferon-γ by neutralizing IL-18 (Hirooka and Nozaki, 2021). In particular, NGAL, Fetuin-A, and IL-18BP are known to induce the expression of pro-inflammatory molecules and activation of various cytokine cascades (Hennige et al., 2008; Jung and Ryu, 2023; Wang, 2024) as presented in **Figure 1**.

Therefore, the purpose of the current investigation was to evaluate the diagnostic potential of the above-described renal injury markers singularly and in combination for the detection of hepatitis B-associated inflammation and very early kidney injury in treatment-naive patients with CHB in various phases of HBV infection.

## Materials and methods

This work is a part of a prospective cross-sectional single-center study. All patients visiting outpatient or inpatient wards of the Department of Gastrointestinal Medicine and Surgical Gastroenterology during the tenure of the study, with complaints like flatulence, fatigue, indigestion, nausea, edema, and a metallic taste in the mouth, were tested for HbsAg positivity. Among these, 109 were found to be HBsAg-positive. Furthermore, 71 patients were confirmed as treatment-naive HBsAg-positive and were considered for this study. The CHB cohort was defined as serum HBsAg positivity for more than 6 months, presence of HBV-DNA, absence of anti-HBc IgM, HCV co-infection, and any associated liver disease. Two patients were excluded due to the serological presence of anti-HBc IgM. A total of 69 age-matched healthy volunteers were considered to determine the normal levels of the biomarkers. Patients with CHB plus HCC, diabetes, or intrinsic renal disease were excluded.

### Sample collection and conventional marker analysis

Overnight fasting blood by venipuncture and mid-stream urine specimens were collected from all participants. The serum was separated from blood samples within 30 min of collection by centrifuging at 3,000 rpm for 15 min. Urine samples were centrifuged at 2,500 rpm for 10 min. Each sample was coded, aliquoted, and stored at −80°C until used for biomarker determination. HBsAg testing was performed using qualitative enzyme-linked immunosorbent assay (ELISA) (Diasorin S.p.A., Saluggia, Vercelli, Italy). At the same time, anti-HBc IgM, HBeAg, and anti-HBeAg were checked using ELISA kits according to the manufacturer’s instructions (Immucheck, Genomix Molecular Diagnostics Pvt. Ltd, India).

HBV DNA quantitation was performed using FRET probes, HBV core-region-specific primers, quantitative standards, and internal controls on real-time PCR (LightCycler 2.0, Roche Diagnostics, Germany; minimum detection limit 10 copies/mL) and recorded from the lab log (Punde et al., 2011).

Serum ALT and AST (Reitman and Frankel, 1957), sCr (Jaffe, 1886), and sUr (Rosenthal, 1955) were evaluated by the enzymatic colorimetric method. The estimated glomerulus filtration rate (eGFR) was calculated using the MDRD equation (modification of diet in renal disease) in mL/min per 1.73 m^2^ (Levey et al., 1999). eGFR = 186 × (sCr) − 1.154× (age) − 0.203 ×0.742 (if the subject is female). Categorization of the patients with CHB was done in accordance with EASL guidelines, 2017 (European Association for the Study of the Liver, 2017), based on HBeAg status, ALT levels, and viral load.

1. HBeAg-positive chronic HBV infection (EPI), previously referred to as immune-tolerant—nine cases with viral load >10^7^ IU/mL and normal ALT.
2. HBeAg-positive CHB (EPB), previously called immune clearance—11 cases with viral load 10^4^–10^7^ IU/mL, moderate to elevated ALT.
3. HBeAg-negative chronic HBV infection (ENI), previously termed as inactive carrier—25 HBeAg-negative and anti-HBe +ve patients with viral titer ≤2 × 10^3^ IU/mL, normal ALT.
4. HBeAg-negative CHB (ENB), previously termed as pre-core mutant—19 HBeAg-negative and anti-HBe positive patients with viral load >2 × 10^3^ IU/mL, moderate to high ALT.

*(Five HBeAg and anti-HBe negative cases were taken to be in the early seroconversion phase and omitted from analysis).

### Biomarker panel for assessing renal dysfunction

Serum levels of Cys-C, NGAL, serum Fetuin-A (sFet-A), and urine content of KIM-1 were analyzed using commercially available ELISAs (R&D Systems, Minneapolis, MN, USA) according to the manufacturer’s instructions. Also, urine levels of IL-18BP were determined using the Raybiotech ELISA kit as per insert (Norcross, GA, USA). The reference range for sCys-C, sNGAL, sFet-A, urinary kidney injury molecule-1 (uKIM-1), and urinary interleukin-18 binding protein (uIL-18BP) was 0–100, 0–10, 0–500, 0–10, and 0–6,000 ng/mL, respectively. The mean minimum detectable dose for each biomarker measured was 0.102 ng/mL for sCys-C, 0.012 ng/mL for sNGAL, 0.062 ng/mL for sFet-A, 0.009 ng/mL for uKIM-1, and less than 20 pg/mL for uIL-18BP. Calibration curves were generated with standards provided for each biomarker using Microplate Manager software 6.1, on iMark, Microplate Reader, and Bio-Rad, and were fit by four-parameter logistic regression.

### Statistical analysis

Descriptive statistics were reported as the median (range) for continuous variables. The analyses were performed using GraphPad Prism software 9.0. Since the data were non-normally distributed, differences between two groups and more than two groups were analyzed using the Mann–Whitney *U* and Kruskal-Wallis tests, respectively. Bivariate correlation analysis was assessed using Spearman’s nonparametric rank correlation coefficients. The receiver operating characteristic (ROC) curve analysis was used to determine the predictive and diagnostic value of the biomarkers. The interactive web tool CombiROC was employed to analyze the diagnostic performance of candidate biomarker panels for the best combinations (Mazzara et al., 2017). Statistical significance was defined by a two-tailed value of *p* < 0.05.

### Ethics statement

The study protocol conforms to the ethical guidelines of the 1975 Declaration of Helsinki (6th revision, 2008). The research procedures were reviewed and approved by the institutional ethics committee (IRB/14/Res/10). Written informed consent was obtained from all participants before inclusion. Permission for publication was received from DG-ICMR.

## Results

### Cohort characteristics

The final analysis for the study was performed on 69 treatment-naive patients with CHB (48 men and 21 women) with a median age of 36 years (19–68) and 69 age-matched healthy individuals (40 men and 29 women). The baseline characteristics of the studied subjects are summarized in **Table 1**. No statistically significant differences between the subjects and controls were observed for conventional renal parameters, sCr and sUr, indicating that they cannot assess the early onset of kidney injury in patients with CHB naive to antiretroviral therapy, thus justifying the selection of our kidney injury marker panel.

**Table 1:** Baseline clinical and biochemical characteristics of subjects under investigation.

Cohort Parameters	Therapy-naive CHB (*n* = 69)	HC (*n* = 69)	*p*-value
Median age (range)	36 (19–68)	35 (19–68)	
Male: Female ratio	48:21	40:29	
BMI (kg/m^2^)	22.17 (16.13–30.14)	22.71 (16.4–28.38)	
HbsAg	Positive	–	
Anti-HBcIgM	Negative	–	
HBeAg	20 positive/49 negative	–	
Anti-HBeAg	63 positive/6 negative	–	
Hypertension	6	–	
ALT (normal range: ≤49 IU/L)	38.5 (17–139)	27 (10–48)	<0.0001****
HBV DNA load log10 (IU/mL)	4.1 (0.52–9.43)	–	–
sCr (mg/dL) (normal range: 0.6–1.4 mg/dL)	0.93 (0.4–1.47)	0.83 (0.58–1.24)	0.56
eGFR mL/min/1.73 m^2^ (normal range: ≥90 mL/min/1.73 m^2^)	90.87 (65.42–145.41)	90.75 (85.31–157.66)	0.54
sUr (mg/dL) (normal range: 10–50 mg/dL)	21 (11–50)	20 (10–38)	0.23
sCys-C (ng/mL)	1,468 (614.6–4,801)	549.1 (297.3–1,409)	<0.0001****
sNGAL (ng/mL)	81.9 (25.64–635)	39.85 (11.78–103.7)	<0.0001****
uIL-18BP (pg/mL)	2,329 (218.5–10,832)	1,326 (536.4–4,700)	<0.0001****
sFetuin A (µg/mL)	642.2 (130.3–9,046)	1,121 (256.6–2,297)	<0.01**
uKIM-1 (ng/mL)	0.468 (0.006–5.85)	0.436 (0.035–1.4)	0.148

Data expressed as median (range). CHB, chronic hepatitis B; healthy controls, HC; BMI, body mass index; HBsAg, hepatitis B surface antigen; anti-HBcIgM, IgM antibody to hepatitis B core antigen; HBeAg, hepatitis B e-antigen; anti-HBeAg, IgM antibody to hepatitis B e-antigen; eGFR, estimated glomerulus filtration rate; sCr, serum creatinine; sUr, serum urea; sCys-C, serum cystatin-C; sNGAL, serum neutrophil gelatinase-associated lipocalin; sFet-A, serum Fetuin-A; uIL-18BP, urinary interleukin-18 binding protein; uKIM-1, urinary kidney injury molecule-1.

*p* < 0.01**, *p* < 0.0001****.

### Markers at baseline

**Figure 2:**
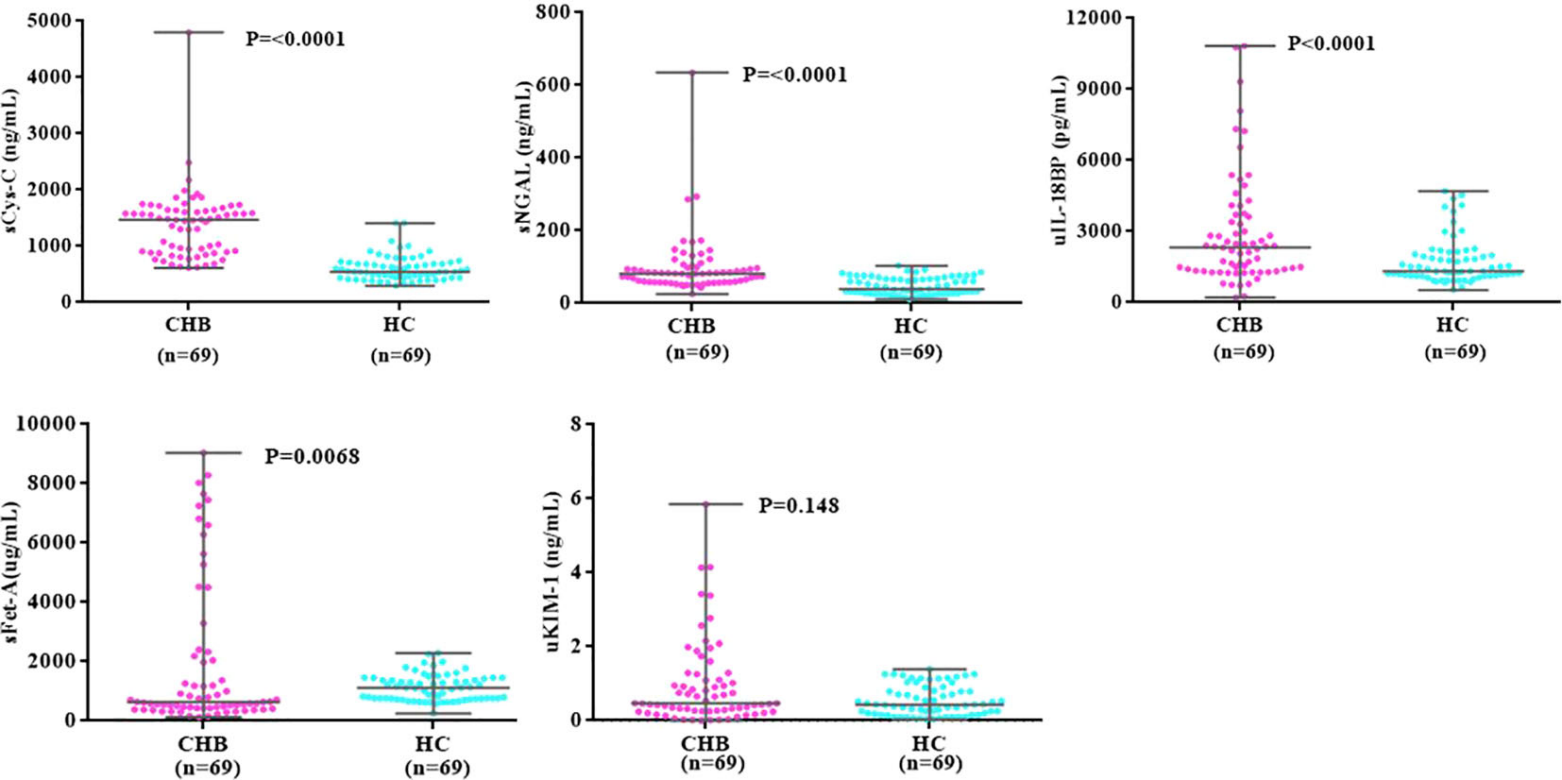
Dot plots comparing baseline levels of markers between CHB and HC groups. CHB, chronic hepatitis B; HC, heathy controls, sCys-C, serum Cystatin-C; sNGAL, serum neutrophil gelatinase associated lipocalin; sFet-A, serum fetuin-A; uIL-18BP, urinary interleukin-18 binding protein; uKIM-1, urinary kidney injury molecule. P-value indicates level of significance.

As shown in **Table 1**; **Figure 2**, the Mann–Whitney test established the baseline levels of sCys-C, sNGAL, and uIL-18BP to be significantly upregulated in the CHB group compared to the healthy controls (*p* < 0.0001, *p* < 0.0001, and *p* = 0.0001, respectively). At the same time, sFet-A was found to be significantly downregulated (*p* = 0.0068). No significant differences were detected in baseline values of uKIM-1 between the analyzed groups.

Next, we deduced the association of sCys, sNGAL, sFet-A, and uIL-18BP in terms of other markers in the panel with conventional renal parameters and viral load using the Spearman rank correlation test. sCys-C showed a very weak positive correlation with uKIM-1 (*r* = 0.2607, *p* = 0.0305) and sCr levels (*r* = 0.24, *p* = 0.047). No correlations emerged for other clinical parameters and kidney biomarkers.

### Sensitivity analyses

**Figure 3:**
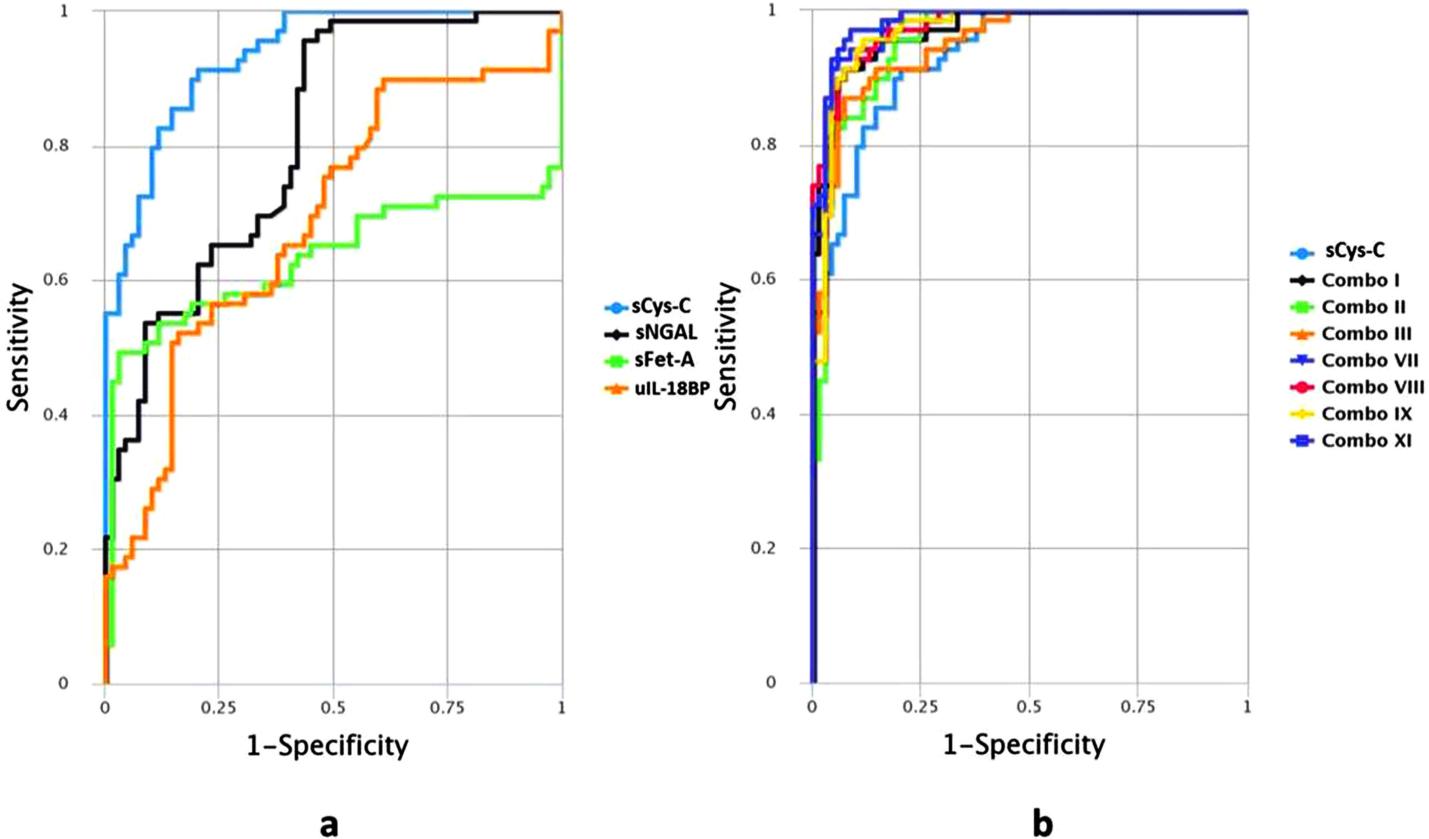
ROC curves presenting diagnostic performance of biomarkers **(a)** individually. sCys-C, serum Cystatin-C; sNGAL, serum Neutrophil gelatinase associated lipocalin; sFet-A, serum Fetuin-A; uIL-18BP, urinary Interleukin-18 Binding Protein. **(b)** as combination panels in comparison with the most robust singular marker sCys-C. sCys-C, serum Cystatin-C, Combo I: sCys-C+sNGAL, Combo II: sCys-C+sFet A, Combo III: sCys-C+uIL18BP, Combo VII: sCys-C+sNGAL+sFet-A, ComboVIII: sCys-C+sNGAL+uIL18BP, Combo IX: sCys-C+sFet-A+uIL18BP, ComboXI: sCys-C+sNGAL +sFet-A+ uIL18BP.

ROC analysis was applied to define the diagnostic performance of sCys C, sNGAL, sFet-A, and uIL-18BP using optimal cutoff points to delineate early kidney injury in patients with CHB (**Table 2**; **Figure 3a**). sCys-C turned out to be the most robust and precise stand-alone indicator, as evident from an AUROC of 0.935 (95% CI, 0.8867–0.9632). At an optimal cutoff value of ≥729.98ng/mL, the sensitivity, specificity, and accuracy were 91.3%, 79.7%, and 85.51%, respectively, indicating accurate differentiation. Next in the line was sNGAL with an AUC of 0.81, 95% CI, 0.7281–0.8704), sensitivity of 95.59% at 56.52% specificity, and accuracy of 75.91%. 

**Table 2:** Receiver operating characteristics and cutoff values of biomarkers for detecting presence of kidney injury in CHB.

Parameter	AUC (95% CI)	*p*-value	Youden index	Cutoffs	Sensitivity	Specificity	Accuracy
sCys-C (ng/mL)	0.935 (0.8867–0.9632)	<0.0001	0.7101	≥729.98	91.30%	79.7%	85.51%
sNGAL (ng/mL)	0.810 (0.7281–0.8704)	<0.0001	0.5211	≥50.10	95.59%	56.52%	75.91%
sFet-A (μg/mL)	0.633 (0.5177–0.7255)	0.0060	0.4638	≤617.89	49.28%	97.1%	73.19%
uIL-18BP (pg/mL)	0.689 (0.5890–0.7686)	0.0001	0.3623	≥2,329.16	50.2%	85.5%	66.89%

*The optimal cutoff value determined by Youden’s index. sCys-C, serum cystatin-C; sNGAL, serum neutrophil gelatinase-associated lipocalin; sFet-A, serum Fetuin-A; uIL-18BP, urinary interleukin-18 binding protein; AUC, area under the curve.

### Biomarker panel analysis

Subsequently, the performance of the above biomarkers together as a panel was evaluated for diagnosing early renal impairment with greater sensitivity and specificity in patients with CHB asymptomatic for any kidney dysfunction. Potential candidate markers were combined in various combinations (two to four proteins) to serve as a panel. Among the 11 possible combinations, 7 outperformed the stand-alone single marker sCys-C in distinguishing HBV-induced kidney dysfunction. The best panel, composed of four proteins (combo XI), presented an AUC value of 0.984 and 97.1% sensitivity at 91.3% specificity. It was followed by combo VII consisting of trio sCys-C+ sNGAL+ sFet-A (AUC=0.980) (**Table 3** and **Figure 3b**).

**Table 3:** Area under the curve for receiver operating characteristics of various combinations for predicting kidney injury in patients with CHB.

Combinations	Markers	AUC	Sensitivity	Specificity
Combo I	sCys C + sNGAL	0.970	0.913	0.928
Combo II	sCys C + sFet A	0.957	0.826	0.957
Combo III	sCys C + uIL-18BP	0.951	0.870	0.928
Combo IV	sNGAL + sFet A	0.813	0.957	0.565
Combo V	sNGAL + IL-18BP	0.823	0.971	0.551
Combo VI	sFet A + IL-18BP	0.689	0.522	0.855
Combo VII	sCys C + sNGAL + sFet A	0.980	0.928	0.957
Combo VIII	sCys C + sNGAL + uIL-18BP	0.976	0.913	0.928
Combo IX	sCys C +sFet A + uIL-18BP	0.969	0.957	0.884
Combo X	sCys C + sFet A + uIL-18BP	0.825	0.971	0.551
Combo XI	sCys C + sNGAL + sFet A + uIL-18BP	0.984	0.971	0.913

sCys-C, serum cystatin-C; sNGAL, serum neutrophil gelatinase-associated lipocalin; sFet-A, serum Fetuin-A; uIL-18BP, urinary interleukin-18 binding protein; AUC, area under the curve.

### Evaluation of different phases of CHB infection

To assess the interplay of viral replication and host immune response in different states of HBV infection within the CHB cohort, detailed analyses between HBeAg-positive and HBeAg-negative subjects were conducted for our markers, but no statistical difference was recorded. However, among various phases of chronic HBV infection (EPI, EPB, ENI, and ENB), a moderately significant difference (*p* = 0.048) was found only in levels of sCys-C as an indicator of kidney dysfunction (**Table 4**), signifying that all HBV infection phases are vulnerable to kidney damage irrespective of replication status. On combining both HBeAg-positive groups (EPI and EPB) as EP, sNGAL levels were seen to differ significantly among EP, ENI, and ENB groups (*p* = 0.039), pointing to an alteration in inflammation status among the patients with active viral replication, carriers, and pre-core mutants. This result indicated a viral infection-initiated host immune response, which may have manifested as inflammation leading to kidney injury during the HBV course of infection.

**Table 4:** Clinical and biochemical characteristics of patients in various phases of HBV infection.

Phases Parameters	EPI (*n* = 9)	EPB (*n* = 11)	ENB (*n* = 25)	ENI (*n* = 19)	*p*-value
Median age (range)	47 (19–74)	49 (23–60)	35 (19–71)	36 (19–67)	
Male: Female ratio	7:2	10:1	18:7	10:9	
BMI (kg/m^2^)	22.58 (18.50–27.754)	22.77 (17.98–29.29)	21.35 (16.13–29.29)	22.18 (16.60–30.14)	
HbsAg	Positive	Positive	Positive	Positive	
Anti-HBcIgM	Negative	Negative	Negative	Negative	
HbeAg	Positive	Positive	Negative	Negative	
Anti-HbeAg	Negative	Negative	Positive	Positive	
HBV DNA load log10 (IU/mL)	8.034 (7.2–9.43)	6.22 (4.63–7.0)	4.1 (3.5–7.75)	2.6 (0.52–3.3)	<0.0001****
ALT (normal range: ≤49 IU/L)	26 (17–44)	51 (39–139)	49 (31–130)	22 (16–45)	<0.0001****
sCr (mg/dL) (normal range: 0.6–1.4 mg/dL)	1.12 (0.51–1.46)	0.89 (0.54–1.2)	0.96 (0.5–1.47)	0.91 (0.5–1.45)	0.24
eGFR mL/min/1.73 m^2.^ (normal range: ≥90 mL/min/1.73 m^2^)	91.8 (65.45–128.89)	93.67 (87.05–106.89)	92.34 (63.38–121.77)	90.22 (71.27–127.73)	0.71
sUr (mg/dL) (normal range: 10–50 mg/dL)	2.72 (0.03–11.66)	21 (18–48)	22 (17–50)	19 (11–28)	0.102
sCys-C (ng/mL)	1,579.31 (754.65–2,175.93)	1,081.31 (624.8–1,752.08)	1,296.64 (614.55–1,930.34)	1,572.62 (771.73–4,800.57)	<0.05*
sNGAL (ng/mL)	83.363 (43.10–635.05)	81.39 (50.76–340)	70.25 (48.01–148.45)	96.74 (51.087–286.17)	0.65
uIL-18BP (pg/mL)	3,715.7 (263.739–10,832)	1,458.33 (218.48–10,772.3)	2,365.14 (724.86–6,557.78)	2,409.73 (753.28–9,328.57)	0.83
sFetuin A (μg/mL)	753.18 (416.02–6,288.05)	474.745 (130.34–6,606.62)	642.229 (294.87–9,045.87)	617.89 (309.07–8,289.09)	0.23
uKIM-1 (ng/mL)	0.425 (0.02–4.136)	0.7 (0.016–5.85)	0.54 (0.03–3.43)	0.46 (0.006–2.77)	0.4

Data expressed as median (range); EPI, HBeAg-positive chronic HBV infection; EPB, HBeAg-positive chronic hepatitis B; ENI, HBeAg-negative chronic HBV infection; ENB HBeAg-negative chronic hepatitis B; BMI, body mass index; HBsAg, hepatitis B surface antigen; anti-HBcIgM, IgM antibody to hepatitis B core antigen; HBeAg, hepatitis B e-antigen; anti-HBeAg, antibody to hepatitis B e-antigen; sALT, serum alanine transaminase; eGFR, estimated glomerulus filtration rate; sCr, serum creatinine; sCys-C, serum cystatin-C; sNGAL, serum neutrophil gelatinase-associated lipocalin; sFet-A, serum Fetuin-A; uIL-18BP, urinary interleukin-18 binding protein; uKIM-1, urinary kidney injury molecule-1.

*p* < 0.05*, *p* < 0.0001****.

When we tried to differentiate within the HBeAg-positive groups (EPB and EPI) as they have similar HBeAg-related pathophysiology, borderline but insignificantly higher sCys-C values (*p* = 0.056) were observed in the EPI group (titer ≥ 10^7^ IU/mL) as compared to EPB, demonstrating increased predisposition for kidney injury in the immune-tolerant phase than those in the immune clearance phase. Correlation analysis also supported the results, pointing to a positive association of sCys-C with viral load (*r* = 0.25, *p* = 0.049) in the EP group.

Similarly, when we analyzed the HBeAg-negative groups (ENB and ENI), significantly elevated levels of sNGAL (*p* = 0.0088), along with borderline insignificance for sCys-C (*p* = 0.052), were observed in the ENI (inactive carrier) phase as compared to the levels in the ENB (pre-core mutant) phase. The analysis was suggestive of a predilection of chronic inactive HBV carriers to develop renal injury, which may be due to their prolonged exposure to HBV infection status without treatment with anti-viral and/or steroids. However, no statistical difference was observed in the baseline values of sFet-A and uKIM-1 in the two-, three-, and four-group comparisons.

### Outcome analysis

At the end of the study on contact follow-up, nine cases out of 69 patients with CHB reported to have developed kidney injury. On tracking back, among these nine cases, five were in an inactive carrier state at baseline, three were in an ENB state, and one was in an EPI state during sampling. All nine cases had increased levels of sCys-C and sNGAL with a median (range) of 1,638.6 (1,004.5–4,800.6) and 99.48 (73.81–171.47), respectively, at baseline. An ROC analysis unveiled an AUC of 0.73 (95% CI, 0.5865 to 0.8820) with 77.78.3% sensitivity and 60% specificity at a cutoff value of ≥1,491 ng/mL for sCys-C, while sNGAL with an AUC of 0.8, 95% CI (0.6773 to 0.9208), and a sensitivity of 77.78% at 76.67% specificity also showed optimal differentiation. Thus, patients with higher baseline sCys-C and sNGAL levels have been observed to present an increased risk of decline in renal function within the 5-year study period compared to subjects with lower baseline sCys-C and sNGAL levels. Log-rank test for disease outcome (early kidney injury) is depicted in **Supplementary Figure 1**.

## Discussion

The inflammatory response generated in renal cells due to host–virus interaction manifests as renal injury in individuals with CHB, contributing to the overall disease burden and increasing mortality risk (Hu et al., 2025). Considerable silent kidney deterioration occurs subclinically before a quantifiable decline in renal function is pathologically diagnosed. Therefore, it is important to identify and treat kidney dysfunction in HBV patients at an early juncture before the symptoms of hepato-renal syndrome impinge.

In routine laboratory practice, sCr is a marker for kidney function estimation. However, sCr does not accurately represent GFR in liver maladies (Griffin et al., 2023) and is also less sensitive in patients with mild renal dysfunction (Qiu et al., 2017). Studies have also propounded that sCr levels and associated GFR values are noticeably biased by non-renal factors such as age, gender, protein intake, and weight of the subjects (Qiu et al., 2017). Similarly, sUr is an unreliable indicator of early kidney dysfunction (Ji et al., 2020). In our present analysis, these traditional renal function parameters were not statistically significant (**Table 1**).

Previous experimental research has explored the role of serum Cys-C, NGAL, and KIM-1 in individuals with liver disease (Kamimura et al., 2018). Nevertheless, as far as we know, this study is the first to investigate the role of these markers in identifying very early asymptomatic renal injury in treatment-naive patients with CHB. When assessed as an indicator of renal dysfunction, circulating sCys-C effectively distinguished between patients with CHB and control subjects (*p* < 0.0001), proving to be a reliable and strong marker, with the highest AUC of 0.935 and a sensitivity of 91.30%. Zheng and his colleagues have also attributed a similar role of sCys-C in renal impairment, specifically in patients with HBV-related fibrosis and cirrhosis (Zheng et al., 2020). Out of the five biomarkers examined, only sCys-C displayed a significant positive correlation with uKIM-1 and sCr, concomitant with the findings of a prior study involving CLD patients (Li et al., 2016).

Prior research has demonstrated that sNGAL is an indicator of early kidney damage in HBV patients undergoing antiretroviral therapy, indicating that the toxicity from prolonged treatment with nucleos(t)ide analogs is responsible for the decline in tubular kidney function (Carey et al., 2018). However, the current evaluation suggests that renal injury is attributed solely to the host’s response to the HBV virus in the absence of any antiretroviral therapy, as the tubular injury protein marker sNGAL (AUC 0.811) was identified to be second most reliable biomarker for detecting renal impairment in HBV patients who have not received antiretroviral treatment.

The third and fourth markers identified were uIL-18BP and sFet-A (**Table 2**; **Figure 3a**). Earlier research has shown that lower levels of sFet-A are associated with early stages of CKD (Deepa and Sasivathanam, 2016), while a recent study using a CKD rat model suggests that sFet-A enhances renal function in CKD due to its anti-inflammatory effects (Ashour et al., 2023). These studies align with our current laboratory findings, which, for the first time, reveal significantly reduced levels of sFet-A in a CHB cohort, indicating inflammation-related renal damage.

Over the past two decades, there have been no human studies on uIL-18BP in patients with CHB with kidney issues. However, a single mention of elevated circulatory IL-18BP was found in subjects with reduced kidney function (Lonnemann et al., 2000). Enhanced expression of uIL-18BP in the present work is plausibly the host’s response to neutralize its inflammatory target IL-18, aligning with the former *in vivo* studies that have demonstrated its ability to inhibit IL-18 (Girard-Guyonvarc’h et al., 2018). The fifth marker, uKIM-1, showed higher baseline levels in the CHB group relative to the control group; however, this difference was statistically insignificant. This finding contradicts earlier research that elucidates increased levels of uKIM-1, attributed to nephrotoxicity caused by antiretroviral therapy in patients with CHB (Li et al., 2016).

**Figure 4:**
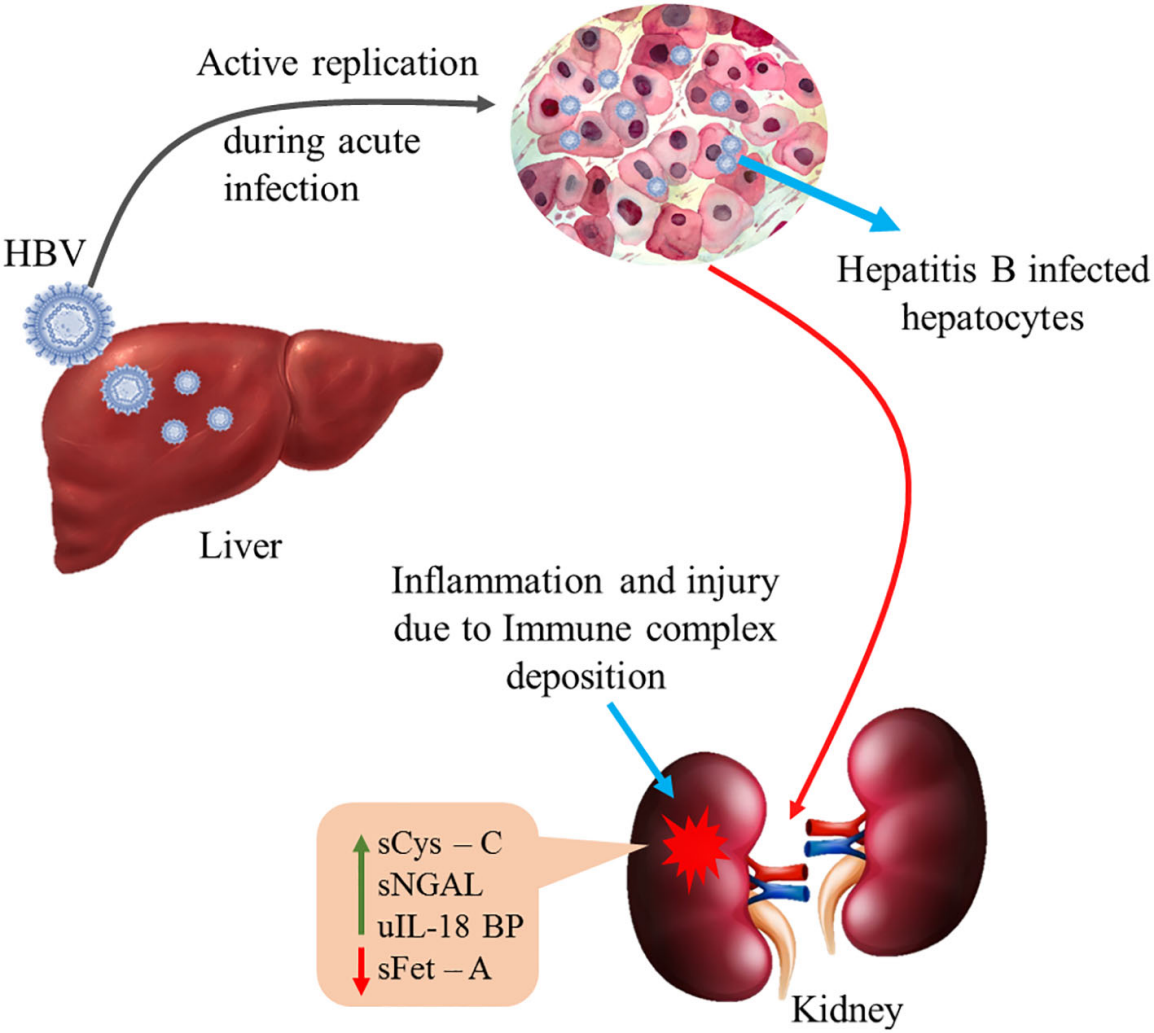
Active HBV replication in the hepatocytes elicits immune response resulting in inflammation and kidney injury due to immune complex deposition. sCys-C, serum cystatin-C; sNGAL, serum neutrophil gelatinase associated lipocalin; sFet-A, serum fetuin-A; uIL-18BP, urinary interleukin-18 binding protein. Green arrow indicates up-regulation of sCys-C, sNGAL and uIL18BP while red arrow indicates down-regulation of sFet-A in circulation.

This is the first attempt where a set of markers—sCysC, sNGAL, sFet-A, and uIL-18BP combined in a panel—have significantly improved AUC and have outshone the efficacy of the singular marker sCysC. Prior work by Prowle and his group has indicated that using a combination of Cr 24 h + post-operative π-GST (π glutathione *S*-transferases) improves AUC (0.86) and is more effective than single markers in identifying renal injury as a comorbidity (Prowle et al., 2015). Our findings regarding sCys-C, sNGAL, sFet-A, and uIL-18BP suggest that these serum and urine biomarkers can offer a distinctive noninvasive approach to screen and distinguish the onset of CHB-related early kidney injury and thereby help to identify patients with CHB anticipated to develop renal insufficiency in the future (**Figure 4**).

The pathogenesis of HBV-mediated nephropathy depends on the complex interplay between the HBV and the host immune response. Regardless of replication status, the serum from patients with CHB can induce apoptotic damage to renal tubular epithelial cells, thereby leading to renal fibrosis (Deng et al., 2006). This *in vitro* finding is consistent with the current study, as even the non-replicative states are equally vulnerable to renal damage. The presence of viral antigens (HBsAg, HBcAg, and HBeAg) and host antibodies expressed in the tubular epithelial cells in response can result in complement activation and apoptosis induction, which have a pivotal role in the pathogenesis of renal injury (Shah and Amarapurkar, 2017). Thus, depending on the phase of infection, HBV antigens, host antibodies, and HBV DNA elicit a host immune response leading to chronic inflammation. Our results have established that sCys-C is very sensitive for detecting early kidney injury, as its values were elevated even in immune-tolerant and inactive HBV carrier phases with normal Cr and ALT. Also, its expression was not associated with liver function markers. This is aligned with Hong and the group’s observation that active HBV replication is not the only factor responsible for the development of kidney disease (Hong et al., 2018). Based on this analysis, an important role for Cys-C as a potential marker of renal function decline is evident. The present investigation also indicates that kidney dysfunction is frequent in our population moderately endemic for HBV, and it occurs even before the initiation of any anti-viral treatment. It emphasizes the need for a baseline renal assessment in all patients with CHB, followed by kidney function monitoring in every phase of HBV infection, irrespective of therapy. However, the conclusions drawn in this investigation are preliminary and need to be validated in a large-sized, multi-centric cohort, which is a limitation of this study.

## Conclusion

In a nutshell, renal oddity in the study is due to host response in the presence of the virus and is pre-existent even before the commencement of antiretroviral treatment. Also, the clinical expediency of the tested markers is remarkable as they can be used not only to screen early renal damage but also to assess the progression of damage noninvasively during the course of HBV infection. The proposed combination (sCys-C + sNGAL + sFet-A + uIL18BP) is even better than sCys-C, a very good stand-alone superior marker compared to the currently used pathological parameters for detecting renal impairment in patients with CHB. However, a larger sample size and follow-up of patients can substantiate the results of our study.

## Data Availability

The raw data supporting the conclusions of this article will be made available by the authors, without undue reservation.

## Supplementary material

Supplementary Figure 1 Kaplan Meier analysis for assessing early kidney injury.
