# Supplementary figures and images for: Detecting early kidney injury due to host–virus interaction response in treatment-naive CHB—a pilot study
Source: Front Cell Infect Microbiol. 2025 Jul 29;15:1601678. doi: 10.3389/fcimb.2025.1601678 (PMC12339531; doi:10.3389/fcimb.2025.1601678)

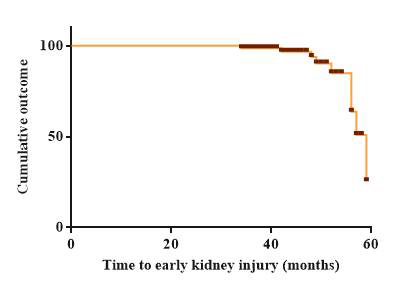

Supplement: Supplementary Figure 1 — Kaplan Meier analysis for assessing early kidney injury. [file Image1.jpeg]
